# Exosome Proteomics Reveals the Deregulation of Coagulation, Complement and Lipid Metabolism Proteins in Gestational Diabetes Mellitus

**DOI:** 10.3390/molecules27175502

**Published:** 2022-08-26

**Authors:** Elena G. Bernea, Viorel I. Suica, Elena Uyy, Aurel Cerveanu-Hogas, Raluca M. Boteanu, Luminita Ivan, Iuliana Ceausu, Doina A. Mihai, Constantin Ionescu-Tîrgoviște, Felicia Antohe

**Affiliations:** 1 “Prof. N. Paulescu” National Institute of Diabetes, Nutrition and Metabolic Diseases, 020474 Bucharest, Romania; 2 Institute of Cellular Biology and Pathology “Nicolae Simionescu”, 050568 Bucharest, Romania; 3 University of Medicine and Pharmacy “Carol Davila”, 020021 Bucharest, Romania; 4 “Dr. I. Cantacuzino” Hospital, 020475 Bucharest, Romania

**Keywords:** gestational diabetes, platelet activation, prothrombotic factors, complement system, lipid metabolism, proteomics, mass spectrometry

## Abstract

Exosomes are small extracellular vesicles with a variable protein cargo in consonance with cell origin and pathophysiological conditions. Gestational diabetes mellitus (GDM) is characterized by different levels of chronic low-grade inflammation and vascular dysfunction; however, there are few data characterizing the serum exosomal protein cargo of GDM patients and associated signaling pathways. Eighteen pregnant women were enrolled in the study: 8 controls (CG) and 10 patients with GDM. Blood samples were collected from patients, for exosomes’ concentration. Protein abundance alterations were demonstrated by relative mass spectrometric analysis and their association with clinical parameters in GDM patients was performed using Pearson’s correlation analysis. The proteomics analysis revealed 78 significantly altered proteins when comparing GDM to CG, related to complement and coagulation cascades, platelet activation, prothrombotic factors and cholesterol metabolism. Down-regulation of Complement C3 (C3), Complement C5 (C5), C4-B (C4B), C4b-binding protein beta chain (C4BPB) and C4b-binding protein alpha chain (C4BPA), and up-regulation of C7, C9 and F12 were found in GDM. Our data indicated significant correlations between factors involved in the pathogenesis of GDM and clinical parameters that may improve the understanding of GDM pathophysiology. Data are available via ProteomeXchange with identifier PXD035673.

## 1. Introduction

Gestational diabetes mellitus (GDM) is a metabolic complication of pregnancy characterized by hyperglycemia and multisystemic dysfunction. It affects ~20% of pregnancies worldwide, with an increasing incidence due to the epidemic nature of obesity among women in the reproductive age [1]. GDM is defined by glucose intolerance with onset during pregnancy and appears when insulin resistance exceeds the capacity of pancreas to secrete insulin. Insulin resistance leads to insulin imbalance and predisposes women with a GDM-complicated pregnancy and their offspring to develop type 2 diabetes mellitus (T2DM) and cardiovascular diseases (CVD) later in life [1,2].

The pathophysiology of GDM is still unclear. Recent studies have been focusing on the role of exosomes in the maternal metabolic adaptative mechanisms during pregnancy associated with GDM. Exosomes are membrane-bound vesicles with a variable protein cargo in consonance with the environmental conditions and cell origin. They are produced by almost every cell type and once released into the extracellular milieu may act locally or at distance, through systemic circulation. Exosomes can be isolated from most of the biological fluids including blood, lymph, saliva, milk and amniotic fluid [3].

It is well-established that exosomes carry molecular contents and influence various physiological factors. An exciting area for research is the role of exosomes in healthy and pathological pregnancies. In inflammatory pathologies, such as SARS-CoV-2, diabetic nephropathy or even GDM, exosome have represented important biological tools in identifying specific molecular content as critical regulators of inflammation, such as drug and anti-inflammatory metabolites, or microRNAs [4,5,6].

Proteomics is widely used in bio-medical research and clinical diagnosis for its ability to analyze, characterize and classify proteins in a proteome [7]. Because proteins secreted into the maternal circulation reflect the physiological or disease state, the pathogenesis of GDM can be further explored using proteomics. This may improve our knowledge of GDM physiopathology. In GDM, a pathogenic vicious circle is generated: a chronic low-grade inflammation generates vascular injury and dysfunction with subsequent platelet activation which lead to an exacerbated vessel injury. A proteomic analysis of plasma collected from pregnant women with GDM and normoglycemia demonstrated that the main differences were related to the coagulation and complement pathways [8]. The complement system plays a role in metabolic disorders and is essential for the adaptive immune response and for the processes that normally appear in pregnancy such as homeostasis of host cells and removal of apoptotic cells. In pregnancies complicated with metabolic disorders, such as GDM, these processes may be dysregulated. Several studies reported that in patients with diabetes-complicated pregnancy, hyperlipidemia increases coagulation activity and hyperglycemia negatively affects coagulation function and lipid metabolism [9,10]. Others reported that in plasma of GDM pregnant women, elevated plasma coagulation activation markers can be identified: factors VII, VIII, XI, XII, fibrinogen, kallikrein, von Willebrand factor and thrombin-antithrombin complex [11]. The underlying molecular mechanisms and pathogenesis of GDM are not well understood but several reports sustain the idea that GDM is a hypercoagulable state, with low-level inflammation and with a high level of thrombotic events. The mean platelet volume (MPV), which can be easily obtained from routine blood analysis, is associated with platelet morphology and activity. Recently, a significant number of studies measured MPV levels in women with GDM in order to evaluate if it can be used to monitor the development of GDM. Unfortunately, the published data regarding the MPV and GDM correlation are contradictory. Several studies showed no association between MPV and GDM [12,13], whereas other reports demonstrated that compared to healthy pregnancies, GDM patients had significantly increased MPV, with the potential to monitor the evolution of GDM [14]. Gorar et al. reported decreased MPV values in patients with GDM [15], while Maluf et al. showed that elevated MPV is also related to T2DM, cardiovascular diseases and nonalcoholic fatty liver disease (NAFLD) [16]. The mechanisms behind the increase of MPV levels in GDM are not yet fully elucidated. Herein, we aimed to reveal the alterations of leading factors and signaling pathways in the serum exosomes of pregnant women with GDM, in the second trimester of pregnancy. We revealed that most differentially abundant proteins found in GDM were related to coagulation function and lipid metabolism.

## 2. Results

### 2.1. Characteristics of the Study Cohort

The age of GDM patients was significantly higher compared with the control subjects (1.21-fold, *p* < 0.001). We measured significantly higher levels of leptin (by 1.58-fold, *p* < 0.05), glucose (at different points: 0, 1 h, 2 h) and triglycerides (by 1.57-fold, *p <* 0.05) in GDM patients vs. controls. The serum levels of Homeostatic Model Assessment for Insulin Resistance (HOMA-IR), HbA1c, creatinine, total cholesterol, high-density lipoprotein (HDL), uric acid, ALT, AST, hemoglobin and glutamic acid decarboxylase autoantibody (antiGAD) were not significantly different. The control group presented similar pre-pregnancy body mass indexes (BMICG = 24.21 ± 5.13 kg/m^2^) compared to diabetic women (BMIGDM = 25.99 ± 5.2 kg/m^2^), although the latter group presented a mean value that would be associated with obesity. There were no significant differences in other clinical parameters including gestational age at delivery, systolic and diastolic blood pressure, and rate of cesarean section or newborn’s anthropometry. All maternal characteristics are presented in Table 1.

### 2.2. Characterization of Serum Exosome Fractions

**Figure 1:**
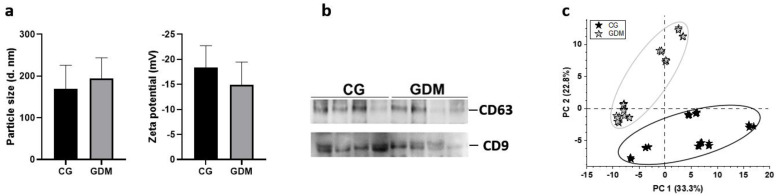
Characterization of exosome fractions concentrated from serum. (**a**) Dynamic light scattering measurements. Size and zeta potential distribution (10 measurements, each) for samples concentrated from pregnant women without (CG) and with diabetes (GDM). (**b**) Detection of exosome markers CD9 and CD63 by Western blot in serum exosome fractions concentrated from CG and GDM. (**c**) Principal component analysis plot of the exosome differentially abundant proteins. A distinct proteome can be discerned in GDM compared with CG group.

Dynamic light scattering assays were performed for characterization of size and stability of concentrated exosome vesicles. In line with previous reports [17], we obtained <200 nm vesicles, with non-significant differences between CG and GDM samples, as observed in Figure 1a. Additionally, a good stability of the nanovesicles was obtained (average Zeta potential of −18.4 for CG samples and −14.9 mV for GDM exosomes), as measured by the Zetasyzer system, after the applied concentration methodology. Representative dynamic light scattering images are provided in the Supplementary Material.

To verify that indeed the concentrated vesicles were of exosomal origin, we pursued the detection of the endosome-specific tetraspanins CD63 and CD9, previously mentioned [18]. Figure 1b highlights that both tetraspanins were expressed in our samples, validating the concentration procedure.

### 2.3. Exosome Proteome Analysis

The proteomic analysis revealed 78 differentially abundant proteins when comparing GDM to CG (>1.25-fold regulation, *p*-value < 0.05). The alteration of proteome specific to the GDM condition was also highlighted by principal component analysis, which demonstrated a spatial separation from the control group (Figure 1c). String bioinformatic mapping of exosome-associated differentially abundant proteins determined statistical associations with complement and coagulation cascades (CCC, pathway code HSA04610, 24 proteins), platelet degranulation (PD, pathway code HSA-114608, 14 proteins) and cholesterol metabolism (CM, pathway code HSA04979, four proteins) pathways and thrombotic diseases-gene associations factors (TF, DOID:2364 and DOID:10772, four proteins), (Table 2).

### 2.4. Differentially Abundant Proteins Involved in the Complement and Coagulation Cascade

**Figure 2:**
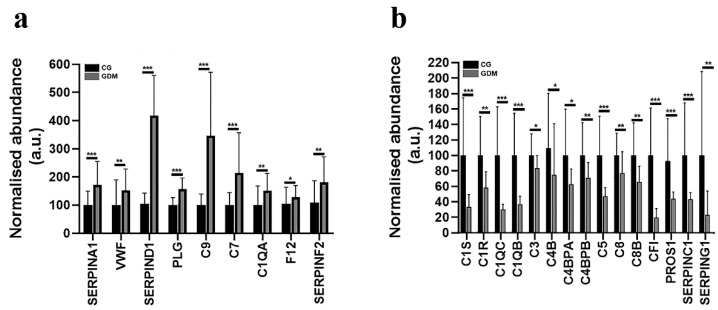
Histograms showing the normalized abundance alterations induced by gestational diabetes for exosomal proteins associated with the complement and coagulation cascade KEGG pathway (HSA04610). (**a**) Up-regulated proteins and (**b**) down-regulated proteins after comparing the spectral abundance of GDM (diabetic gestational group) to CG (control group). Data represent the mean ± SD; * *p* ˂ 0.05; ** *p* < 0.01; *** *p* < 0.001.

In the case of GDM exosomes, our data reflect an altered spectral abundance for twenty-four proteins correlating with the CCC signaling pathway with high statistical confidence (FDR *p*-value = 1.44e−33). These proteins are: Alpha-1-antitrypsin (SERPINA1), Von Willebrand factor (VWF), Heparin cofactor 2 (SERPIND1), Coagulation factor XII (F12), Plasma kallikrein (KLKB1), Plasminogen (PLG), Antithrombin-III (SERPINC1), Alpha-2-antiplasmin (SERPINF2), Vitronectin (VTN), Complement component C6 (C6), Complement component C9 (C9), Vitamin K-dependent protein S (PROS1), Complement C3 (C3), Complement component C7 (C7), Plasma protease C1 inhibitor (SERPING1), Complement component C8 beta chain (C8B), Complement C5 (C5), Complement C1q subcomponent subunit B (C1QB), Complement factor I (CFI), Complement C4-B (C4B), C4b-binding protein beta chain (C4BPB), C4b-binding protein alpha chain (C4BPA), Complement C1s subcomponent (C1S), Complement C1q subcomponent subunit A (C1QA), Complement C1r subcomponent (C1R), Complement C1q subcomponent subunit C (C1QC) (Figure 2a,b).

Nine proteins involved in the CCC signaling pathway were found to be significantly upregulated in all GDM samples when compared to controls (Figure 2a): SERPINA1 (by 2.1-fold, *p* ≤ 0.001), VWF (by 1.95-fold, *p* ≤ 0.01), SERPIND1 (by 4.36-fold, *p* ≤ 0.001), PLG (by 1.82-fold, *p* ≤ 0.001), C9 (by 2.22-fold, *p* ≤ 0.001), C7 (by 1.44-fold, *p* ≤ 0.001), C1QA (by 2.17-fold, *p* ≤ 0.01), F12 (by 1.67-fold, *p* ≤ 0.05) and SERPINF2 (by 1.74-fold, *p* ≤ 0.01). The LC-MS/MS analysis also revealed that the spectral abundances of the following proteins were significantly down-regulated: C1S (by 4.07-fold, *p* ≤ 0.001), C1R (by 2.14-fold, *p* ≤ 0.01), C1QC (by 3.22-fold, *p* ≤ 0.001), C1QB (by 3.61-fold, *p* ≤ 0.001), C3 (by 1.25-fold, *p* ≤ 0.05), C4B (by 1.36-fold, *p* ≤ 0.05), C4BPA (by 1.3-fold, *p* ≤ 0.05), C4BPB (by 1.27-fold, *p* ≤ 0.01), C5 (by 2.22-fold, *p* ≤ 0.001), C6 (by 1.58-fold, *p* ≤ 0.01), C8B (by 1.84-fold, *p* ≤ 0.01), CFI (by 6.54-fold, *p* ≤ 0.001), PROS1 (by 1.97-fold, *p* ≤ 0.001), SERPINC1 (by 1.85-fold, *p* ≤ 0.001) and SERPING1 (by 7.04-fold, *p* ≤ 0.01) in the GDM reported to CG group (Figure 2b). Four of the proteins were also found to be involved in local acute inflammatory response (Wikipathway WP4493, FDR *p*-value = 9.89e−05): C7, C3, C5 and C6.

### 2.5. Differentially Abundant Proteins Involved in Platelet Degranulation

**Figure 3:**
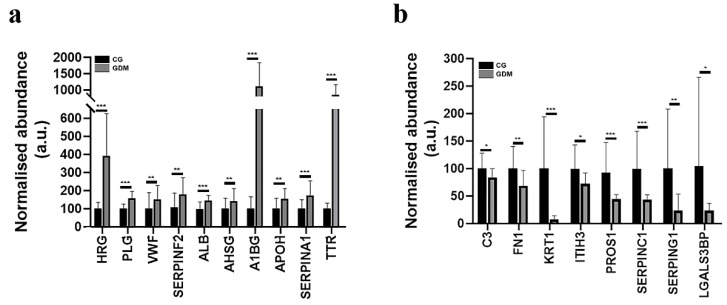
Histograms demonstrating the normalized abundance alterations induced by gestational diabetes for exosomal proteins associated with the Platelet degranulation pathway. (**a**) Up-regulated proteins and (**b**) down-regulated proteins after comparing the spectral abundance of GDM (diabetic gestational group) to CG (control group). Data are expressed as means ± standard deviation (SD); * *p* ˂ 0.05, ** *p* ˂ 0.01; *** *p* ˂ 0.001.

In the GDM exosomes, we detected an altered spectral abundance for fourteen proteins, which strongly correlate with the platelet degranulation signaling pathway (PD, FDR *p*-value = 1.7e−14). These are: Vitamin K-dependent protein S (PROS1), Histidine-rich glycoprotein (HRG), Inter-alpha-trypsin inhibitor heavy chain H3 (ITIH3), Plasminogen (PLG), Plasma protease C1 inhibitor (SERPING1), VWF, Alpha-2-antiplasmin (SERPINF2), serum albumin (ALB), Galectin-3-binding protein (LGALS3BP), Fibronectin type III domain containing (FN1), Alpha-2-HS-glycoprotein (AHSG), Alpha-1B-glycoprotein (A1BG), Beta-2-glycoprotein 1 (APOH), Alpha-1-antitrypsin (SERPINA1) (Figure 3a,b). Our study also detected eight proteins associated with Blood platelet pathway [BTO:0000132]: C3, SerpinC1 (Antithrombin-III), AHSG (Alpha-2-HS-glycoprotein), VWF, ALB, TTR (Transthyretin), KRT1 (Keratin, type II cytoskeletal 1), SERPINA1 (Alpha-1-antitrypsin) (Figure 3a,b).

### 2.6. Gestational Diabetes Regulates Proteins Involved in the Post-Thrombotic Syndrome and Cholesterol Metabolism

**Figure 4:**
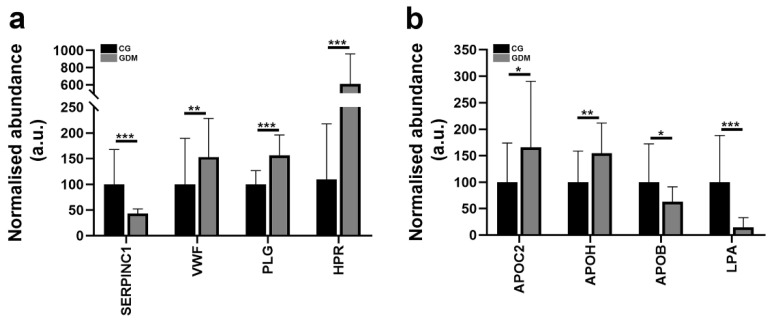
Histograms showing the normalized abundance changes induced by diabetes for (**a**) prothrombotic factors (two proteins associated with post-thrombotic syndrome: antithrombin-III, plasminogen and two proteins associated with thrombotic thrombocytopenic purpura: Haptoglobin and von Willebrand factor) from serum exosomes fractions concentrated from control (CG) and diabetic gestational group (GDM) and (**b**) proteins implicated in cholesterol metabolism KEGG pathway (HSA04979) from the serum exosome proteins concentrated from CG and GDM group. Data represent the means ± SD; * *p* <0.05; ** *p* < 0.01; *** *p* < 0.001.

The proteomic analysis evidenced four prothrombotic factors altered at protein levels by diabetes in the serum exosomes fractions when compared to the control samples. The string analysis of the differentially abundant proteins according to the disease section revealed two of them were associated with post-thrombotic syndrome (FDR *p*-value = 0.0101): antithrombin-III, plasminogen and two with thrombotic thrombocytopenic purpura (FDR *p*-value = 0.0127): haptoglobin and von Willebrand factor (Figure 4a). The proteins involved in the cholesterol metabolism that were found to be differentially abundant were: APOC2 Apolipoprotein C-II; APOH Beta-2-glycoprotein 1; APOB Apolipoprotein B-100; LPA Apolipoprotein(a) (Figure 4b).

### 2.7. Alternative Validation Studies

**Figure 5:**
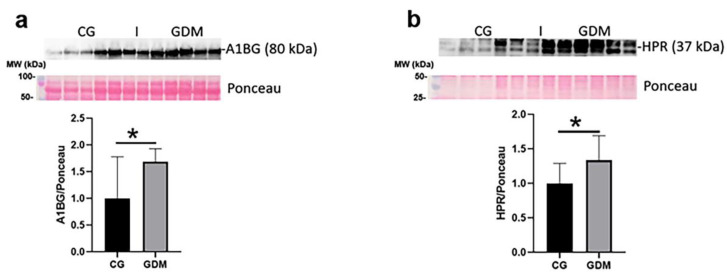
Validation by Western blotting of some protein markers identified by mass spectrometry: (**a**). Alpha-1-B Glycoprotein (A1BG) and (**b**). Haptoglobin-related protein (HPR). Representative immunoblots and densitometric analysis for each protein are shown. Ponceau S staining was used for normalization of the proteins’ levels. Western blot analysis of serum exosome lysates demonstrated protein level alterations in A1BG and HPR in the GDM vs. CG comparison. Statistical analysis was performed using unpaired Student’s *t* test, and the results were expressed as means ± standard deviation (SD); * *p* ˂ 0.05. CG: control group (*n* = 6) and GDM: gestational diabetes group (*n* = 6).

Mass spectrometry data were further validated by an alternative, immune-based methodology (i.e., Western blot). Notably, the mass spectral abundance alteration of A1BG (up-regulation by 1.7-fold and *p <* 0.05) and HPR (up-regulation by 1.3-fold and *p <* 0.05) were confirmed by immunoblotting of serum exosome lysate concentrated from GDM and CG groups (Figure 5).

### 2.8. Statistical Correlations between Serum Clinical Parameters and Dysregulated Proteins

**Figure 6:**
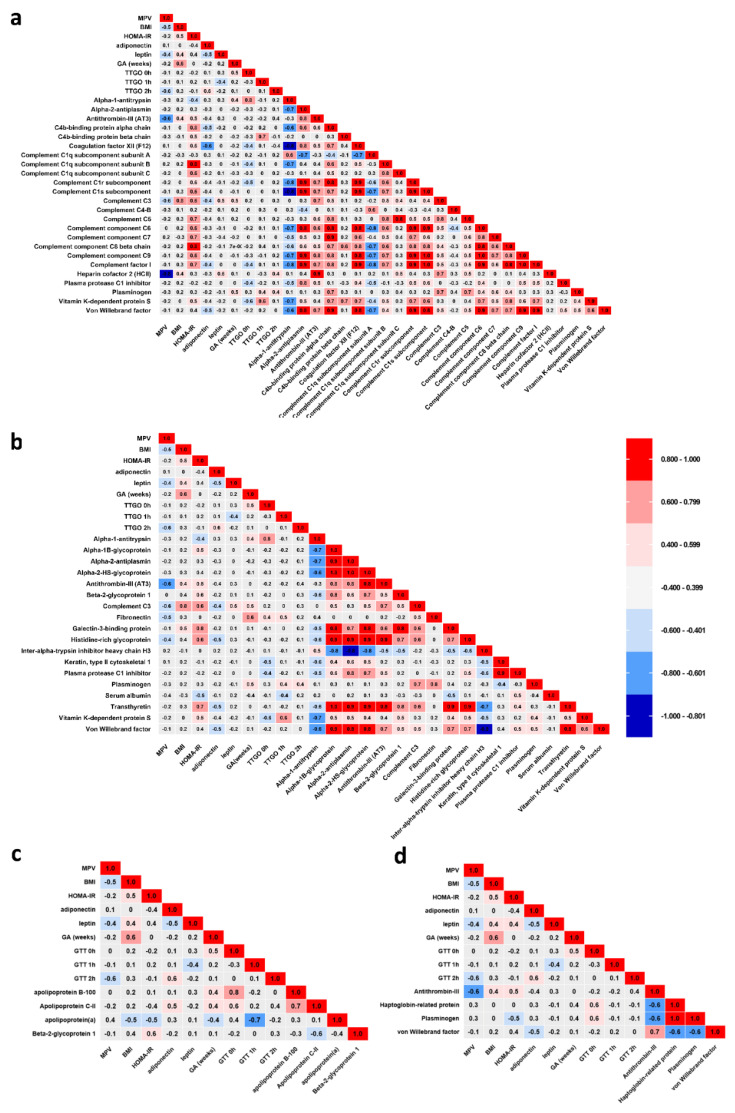
Illustrations showing the associations after applying the statistical Pearson correlation test; (**a**) correlations between parameters and protein molecules belonging to the complement and coagulation cascade signaling pathway; (**b**) correlations between the clinical parameters and proteins involved in platelet degranulation pathway; (**c**) correlations between serum parameters and proteins involved in cholesterol metabolism signaling pathway and (**d**) correlations for prothrombotic factor proteins and clinical parameters. Pearson correlation coefficient is presented from 1 (most significant positive correlation) to −1 (most significant negative correlation).

The spectral abundance of alpha-1-antitrypsin (SERPINA1) was positively correlated with serum glucose at time 0 (GTT 0 h) (r = 0.8, *p <* 0.05) and negatively correlated with HOMA-IR (r = −0.4, *p <* 0.05) in GDM group (Figure 6a). We also found that C3 positively correlated with BMI (r *=* 0.8, *p <* 0.05), HOMA-IR (r *=* 0.6, *p <* 0.04), AT III (r *=* 0.7, *p <* 0.05), and negatively with MPV (r *=* −0.6, *p <* 0.02) and adiponectin (r *=* −0.4, *p <* 0.05). C4BP presented a positive correlation with F12 (r *=* 0.8, *p <* 0.05). An interesting observation is that HOMA-IR correlated positively with many complement factors: C5 (r *=* 0.7, *p <* 0.05), C6 (r *=* 0.6, *p <* 0.05), C7 (r *=* 0.7, *p <* 0.05), C8BC (r *=* 0.8, *p <* 0.05), C9 (r *=* 0.6, *p <* 0.05), CF1 (r *=* 0.7, *p <* 0.05) (Figure 6a). F12 is positively correlated with HOMA-IR (r *=* 0.6, *p <* 0.04), antiplasmin (r *=* 0.8, *p <* 0.05) and C4BP alpha chain (r *=* 0.7, *p <* 0.050 and negatively correlated with adiponectin (r *=* −0.6, *p =* 0.02), serum glucose at time 0 (GTT 0 h) (r *=* −0.4, *p <* 0.05) and SERPINA1 (r *=* −0.8, *p <* 0.05) (Figure 6a). MPV correlated negatively with AT III (r *=* −0.6, *p <* 0.02), C3 (r *=* −0.6, *p <* 0.04), fibronectin (r *=* −0.5, *p <* 0.02) (Figure 6b).

Apo-CII is positively correlated with adiponectin (r *=* 0.5, *p =* 0.05), serum glucose at time 0 (GTT 0h), (r *=* 0.5, *p =* 0.04), apo B100 (r *=* 0.7, *p =* 0.01) (Figure 6c). We also found significant Pearson correlations between apo-CII and: alpha 1 antitrypsin (r *=* 0.8, *p =* 0.002), alpha 2 antiplasmin (r *=* −0.6, *p =* 0.01), F12 (r *=* −0.8, *p =* 0.0009), C1q subunit A (r *=* 0.5, *p =* 0.04), C1s (r *=* −0.73, *p =* 0.0074), C6 (r *=* −0.56, *p =* 0.043), C9 (r *=* −0.6, *p =* 0.02), complement factor I (CFI) (r *=* −0.7, *p =* 0.01) (Figure 6c). Apo B100 is positively correlated with serum glucose at time 0 (GTT 0h), (r *=* 0.7, *p =* 0.004) (Figure 6c).

vWF, the representative endothelial biomarker, is positively correlated with alpha 2 antiplasmin (r *=* 0.8, *p <* 0.05), AT III (r *=* 0.7, *p <* 0.05) (Figure 6a) which is positively interrelated with HOMA-IR (r *=* 0.5, *p <* 0.05), alpha 1 glycoprotein (r *=* 0.9, *p <* 0.05), alpha 2 antiplasmin (r *=* 0.8, *p <* 0.001), alpha 2 HS glycoprotein (r *=* 0.8, *p <* 0.05), and negatively correlated with MPV (r *=* −0.6, *p <* 0.02) (Figure 6d).

## 3. Discussion

Our proteomic analysis revealed specific alterations in the protein exosomal cargo associated with patients with GDM when compared to control subjects. Using high performance mass spectrometry, we identified that most exosomal proteins which were differentially regulated by GDM were primarily associated with complement and coagulation cascades, platelet activation, prothrombotic factors and cholesterol metabolism. The data may be valuable in elucidating the underlying physiological mechanisms associated with GDM. Previously, Jayabalan et al. have successfully applied data-independent mass spectrometric analysis to characterize systemic exosomal proteins and their regulation in GDM patients [19]. Using sophisticated bioinformatic tools, the authors identified a set of differentially abundant proteins regulated by GDM related to energy production, nucleic acid metabolism or hematological or inflammatory disease and so on. The study revealed molecules, most notably calcium/calmodulin dependent protein kinase II beta and Pappalysin-1, among others, as potential markers in the augmentation of GDM-dependent inflammatory response.

Herein, we identified in the exosomes concentrated from the GDM group, twenty-four differentially abundant proteins that were correlated with the CCC signaling pathway with high statistical confidence. To our knowledge, this is the first time the alteration of CCC signaling pathway has been revealed in the serum exosomes of GDM patients. Ramanjaneya et al. reported the first plasma analysis of all three complement system cascades (classical, alternative and lectin pathway) in GDM pregnancies [20]. The complement system plays an important role in inflammation, and its activation is initiated via three different pathways: the classic pathway, the alternative pathway and the lectin pathway. In normal pregnancy, activation of the complement system leads to elevated plasma concentrations of C3a, C4a and C5a. This mechanism appears in order to counterbalance the normal suppression of adaptive immunity [21]. Studies showed that disorders of complement regulation are associated with gestational diabetes and preeclampsia [22]. GDM is a pro-inflammatory state characterized by increased oxidative stress secondary to insulin resistance. These processes lead to higher C3 levels [23]. In our study, in concentrated exosomes from pregnancy complicated with GDM, we found decreased levels of Complement C3 (C3), Complement C5 (C5), C4-B (C4B), C4b-binding protein beta chain (C4BPB), and C4b-binding protein alpha chain (C4BPA). We further analyzed Complement C3 using statistical correlation test in women with GDM. We obtained a statistical negative correlation between Complement C3 and MPV and also between Complement C3 and adiponectin. Moreover, we observed strong and positive correlation with BMI, HOMA-IR and AT III. Our results are sustained by published data but our study is the first one to reveal the dysregulation of complement C3 in GDM maternal serum exosomes. Other studies reported that maternal plasma C3 levels are significantly and positively correlated with maternal markers of insulin and lipid metabolism. Women with GDM and high BMI have higher C3 levels compared to those with normal glucose tolerance and serum C3 levels are associated with body fat mass [24,25]. To our knowledge, the association between maternal serum exosomes of C3 levels and obesity and insulin resistance has not yet been investigated in GDM pregnancies. We have therefore examined this in detail in GDM patients, which present a relatively high risk of type 2 diabetes and subsequent CVD. Serum Complement factor 3 (C3) is a risk factor in cardiovascular diseases (CVD) and obesity-related metabolic diseases. Obesity is defined as a low-grade inflammation that is often accompanied by insulin resistance. We noticed that insulin resistance is strongly associated with serum exosomal C3 levels of GDM pregnancies. Our results are sustained by other studies which showed that HOMA-IR is a strong mediator between obesity and serum C3 levels [26,27]. In addition, we found an association between serum adiponectin, which is a marker of obesity and insulin resistance, with serum exosomes C3 levels in GDM pregnancies. Studies demonstrated that dysregulated serum C3 levels are associated with incidence of type 2 diabetes mellitus, myocardial infarction and hypertension [28,29]. Wlazlo et al. demonstrated that plasma C3 levels reveal a metabolic dysregulation that induce insulin resistance progression and eventually leading to type 2 diabetes mellitus [30]. Future studies are necessary to further elucidate these mechanisms. We also found multiple correlations between complement C3, complement C4 and other complement proteins, reflecting their closely integrated regulation.

In 2022, a systematic review and meta-analysis of biomarkers that are differentially expressed in women with and without GDM revealed that in GDM, C6, C7, C8B, C8G, C9 and C4BPA are downregulated [31]. It is important to note that this recent report mentioned that there are not case-control studies performed with sample collection from serum exosomes. In the present study, we detected that in serum exosomes concentrated from GDM subjects, C4BPA, C6 and C8B are downregulated (similar results to the report above) but, in contrast, we found that C7 and C9 are upregulated. C6, C7, C8B, C8G and C9 are the final complement components, essential in creating the membrane attack complex, which is the main effector of complement-mediated tissue damage [23]. Our data reveal that F12 is upregulated and confirms previous results [31]. Women with GDM have higher serum exosomal levels of F12 when compared to women with healthy pregnancies. There is little information regarding the role of F12 in GDM and, to our knowledge, there are not any studies specifically conducted on serum exosomal levels that report the alteration of F12 levels in GDM. Factor 12 is part of the normal coagulation system and its level increases in pregnant women with gestation progression [32]. F12 is strongly related to kallikrein-kinin system, which is activated by hyperglycemia in uncontrolled diabetes, which increases the level of F12 [11]. There is information suggesting that F12 regulates the growth factor-like (EGF- like) activity through an EGF- like domain which can stimulate liver growth and increase protein production in the liver. These processes lead to the production of F12 and possibly other coagulation factors. Schmeidler-Sapiro et al. suggest that this growth factor-like activity of F12 can be the underlying mechanism that induces hypercoagulability and adverse outcomes in GDM such as preeclampsia and macrosomia [33]. Further studies are necessary in this direction to reveal the signaling pathways that should be targeted by adequate therapeutic medication.

SERPINA1 (or alpha-1-antitrypsin) is encoded in humans by the SERPINA1 gene and is one of the most abundant serine protease inhibitors secreted by the liver. There are contradictory data concerning the level of SERPINA1 in diabetic subjects. Several studies indicated increased levels of alpha-1-antitrypsin in the serum of diabetic subjects, while others showed in fact decreased serum concentrations of SERPINA1 and increased plasma levels in insulin dependent diabetes [34,35]. Lisowska-Myjak et al. reported that during normal and diabetic pregnancy serum alpha-1-antitrypsin increases and its concentration in the serum of pregnant women with diabetes does not depend on the value of glycemic control [36]. Another study showed that concentration of serum alpha-1-antitrypsin is significantly lower in GDM compared to healthy pregnant women [37]. To our knowledge, the present study is the first report to analyze SERPINA1 in the serum exosomes of GDM patients. Our results demonstrate that alpha-1-antitrypsin is upregulated in GDM and is positively correlated with serum glucose at time 0 (GTT Oh). The clinical significance of these results is unclear. There are several studies that have suggested that SERPINA1 has antidiabetic effects, an idea sustained by the anti-inflammatory properties and the ability to protect pancreatic β cells from apoptosis [38,39,40]. More studies in this direction might help in elucidating a potential alpha-1-antitrypsin-based treatment in GDM.

Pathogenesis of GDM and that of type 2 diabetes are similar and the investigation of the interconnection between these two pathological states is based on morphological changes of platelets. The idea that platelets’ activity plays a role in pathogenesis of type 2 diabetes was demonstrated in several studies which showed that MPV values increase in diabetic patients compared with nondiabetic patients [41]. MPV is a useful and cost-effective marker that can be used to evaluate platelet morphology. Increased MPV reflects thrombocyte synthesis, activation, aggregation and adhesion. Rao AK. et al. suggested that a large volume of platelets increases the procoagulant activity in diabetic subjects [42]. Elevated MPV is associated also with increased platelet aggregation and increased thromboxane A2 [43]. As was observed in type 2 diabetes, the insulin resistance and hyperglycemia might be the principal underlying mechanism of the changes in platelet morphology and functions in GDM. There are several studies which investigated the association between MPV and GDM. Bozkurt et al. showed significantly increased MPV values in patients with GDM in comparison to a control group and Erikçi et al. reinforced these findings [44,45]. Yin et al. reported similar levels of MPV in healthy pregnant women and those with GDM [46]. Data are contradictory about this correlation. We therefore aimed to look further into the relationship between MPV and GDM. Thus, in our experimental setting, the mean MPV value of GDM patients was slightly higher than that of control group, albeit non-significantly. There are studies that suggested that increased MPV values are correlated with risk factors for cardiovascular diseases such as impaired fasting blood glucose, insulin resistance, diabetes mellitus, hypertension, hyperlipidemia and metabolic syndrome [47,48]. Among the clinical and biochemical parameters that we collected, we found a negative statistical correlation between MPV and BMI, MPV and leptin, which are markers of obesity and insulin resistance. Additionally, we observed that MPV values were negatively correlated with GTT 2 h. Several studies showed that MPV is an important biological variable which predicts risk of vascular complications in diabetes such as the prothrombotic state [49]. In diabetic patients, the process of platelet-dependent thrombin formation is increased and fibrinolysis decreases [50]. These observations are sustained by our results. In our study, we observed that, in GDM patients, antithrombin III is downregulated and alpha-2-antiplasmin is upregulated. Although this process is not yet fully understood [51], the main factors that lead to hypofibrinolysis in diabetes are a dysfunctional fibrinolytic system and altered fibrin structure. In diabetes, the structure of fibrin clots is more compact and more resistant to fibrinolysis. Impaired fibrinolysis is the result of antifibrinolytic proteins such as complement C3 and plasmin inhibitor [20]. Our results point to a negative correlation of MPV with markers of CCC identified in serum exosomes of GDM pregnancies (antithrombin III and complement C3). This is the first time these correlations have been performed. Other studies showed that increased MPV and enhanced aggregation capacity lead to a pro-thrombotic state [52]. It is also demonstrated that in diabetic pregnant women with poor glycemic control, MPV is an easily measurable parameter that can predict a thrombotic state [49]. In patients with diabetes, over-activation of platelets plays an important role in pro-thrombotic events [53]. Our proteomic analysis evidenced four prothrombotic factors that were altered by GDM in the serum exosome fractions: antithrombin-III, plasminogen, haptoglobin and von Willebrand factor. von Willebrand Factor (VWF) is an abundant plasma glycoprotein which adheres at vascular sites with injury, where it recruits platelets. Variations in VWF levels are clinically significant because high levels of VWF are associated with thrombosis. AT III is an important physiological anticoagulant plasma factor which keeps the balance between coagulation and anticoagulation. A decreased level will indicate an injury of vascular endothelial cells. In patients with GDM, an elevated expression of VWF and reduced expression of AT in plasma suggest endothelial injury and endothelial dysfunction [54]. Our results sustain these observations, demonstrating upregulated VWF serum exosomal levels in GDM vs. control. Studies suggest that damaged endothelial cells generate a vicious circle and promote more GDM development and the abnormal changes of VWF and AT III can be useful indicators of GDM and vascular endothelial cell injury [54]. Given the high risk of development cardiovascular diseases later in life, understanding these mechanisms that appear in GDM is of great importance.

It has been previously demonstrated that changes in the levels of apolipoprotein C-II (apoC-II) and apolipoprotein C-III (apoC-III) may be involved in the mechanisms underlying dyslipidemia in insulin resistance conditions. ApoC-II is an activator of lipoprotein lipase and increased levels of total apoC-II appear in patients with type 2 diabetes [55]. We highlight that serum exosomal levels of apoC-II, which have not been previously reported, were higher in GDM pregnancies compared to normal pregnancies. Recently, Kopylov et al. analyzed the molecular pathophysiology of diabetes mellitus during pregnancy with antenatal complications [56]. They performed proteomic analyses of whole peripheral blood and umbilical blood samples and observed that apoC-II levels diminished by 20–30% in groups of GDM patients. In our case, we observed that serum exosomal levels of apoC-II were positively correlated with adiponectin and fasting glycemia. GDM is considered an insulin resistance condition characterized by decreased levels of adiponectin. Therefore, our results sustain the important role of apoC-II in dysregulation of lipid metabolism in insulin resistance states. Moreover, in our studies, we observed that serum exosomal levels of apoC-II are strongly and positively correlated with ApoB100, which mediates the cholesterol clearance, the transport of LDL and also the exocytosis of lysosomal and peroxisomal proteins [57]. Moreover, Kopylov et al. showed that apo B 100 affects the activity of the complement system [56]. Other evidence suggests the existence of a crosstalk between metabolic organs and complement system in the course of metabolic disorders such as metabolic dysregulation of the adipose tissue, of the liver and the pancreas [58]. All these conditions are related to the development of insulin resistance and diabetes mellitus. In our study we observed that apoB was down-regulated in the serum exosomes of GDM subjects. Applying the statistical Pearson correlation test, we found that numerous complement proteins were positively associated with apoC-II protein. Thus, apoC-II was positively associated with complement C1q subcomponent subunit A, complement C1q subcomponent subunit B, complement C1s, complement component C6, complement C9, F12 and complement factor I. This is the first time correlation between apoC-II and complement factors has been performed.

There is evidence demonstrating that lipoproteins can induce the activation of complement system and consequently the opsonization and binding of these complexes to complement receptors [59] Published data revealed that both native LDL and acLDL induced complement activation with subsequent C3b opsonization. We also found that apo CII correlates positively with complement C1q and other complement components but we cannot draw a conclusion about the relationship between apoC-II and the complement system. Perhaps, apo CII, as other lipoproteins, is involved in the activation of the complement system, but future studies are necessary in order to elucidate the underlying mechanisms.

## 4. Materials and Methods

### 4.1. Study Subjects

**Figure 7:**
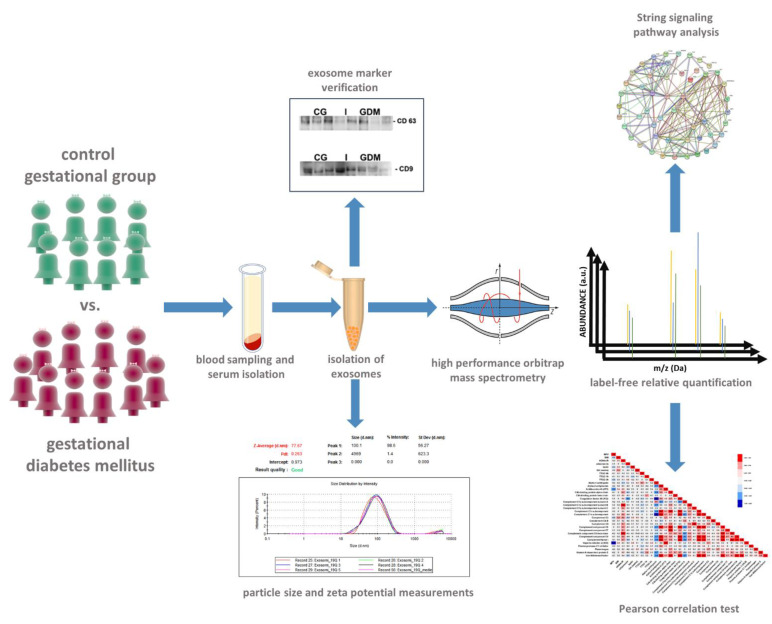
Experimental workflow and methodological approaches. The patients were divided into two groups based on oral glucose tolerance test (GTT) at 0 h, 1 h and 2 h during the third trimester of pregnancy: 8 were considered the control gestational group (CG), while 10 patients formed the gestational diabetes mellitus group (GDM). Blood was drawn from each patient and control subject for serum isolation, exosome enrichment and subsequent proteomic analyses. Label-free relative quantification was followed by bioinformatic pathway analysis. Pearson correlation was pursued to associate the regulation trend of the differentially abundant molecules with clinical and paraclinical variables.

We designed a case-control study which included 18 pregnant women, 8 forming the control group (CG), while 10 patients presented gestational diabetes mellitus (GDM). The study compared the exosomal protein cargo between GDM patients and healthy pregnant women with normal glucose tolerance. In the 3rd trimester of gestation, serum samples were collected from all patients for biochemical, immunological and proteomic analyses (Figure 7).

GDM was diagnosed by 75 g oral glucose tolerance test (GTT) at two-hours as recommended by National Institute for Health and Care Excellence (NIHCE), American Diabetes Association (ADA) [2]. GDM was diagnosed if one or more of the glucose level values exceeded the cut-off limit: fasting ≥ 92 mg/dL, 1 h ≥ 180 mg/dL, 2 h ≥ 153 mg/dL. Patient exclusion criteria were based on age (<18 years and > 40 years) and presence of hypertension, nephropathy, preeclampsia, retinopathy and psychiatric treatment.

### 4.2. Sociodemographic Data

We analyzed pre-pregnancy body weight, body mass index (BMI), anthropometric measurement height and blood pressure. Sociodemographic data of the subjects including parity, smoking status, family medical history, maternal age and socioeconomic status were obtained by anamnesis.

### 4.3. Blood Sampling, Serum Parameters Measurement and Exosome Concentration and Characterisation

All blood samples were collected after at least 8 h of fasting. The samples were centrifuged at 1000× *g* for 15 min and stored at –80 °C until further use. The levels of serum adiponectin, C-peptide, insulin, leptin and proinsulin were measured by an enzyme-linked immunosorbent assay (ELISA) EIA-4177, EIA-1293, EIA-2935, EIA-2393 and EIA-1560, respectively (DRG International Inc., Springfield, IL, USA) using the manufacturer’s recommendations. Serum cholesterol, HDL and triglyceride level determinations were performed using commercially available kits from DIALAB GmbH (Vienna, Austria). Fasting glucose was determined by the glucose oxidase method using a glucose analyzer (Beckman Instruments, Fullerton, CA, USA), while glycated hemoglobin (HbA1c) levels were determined by Variant II Turbo HbA1c analyzer (Bio-Rad Laboratories, Hercules, CA, USA). Serum levels of creatinine, uric acid, alanine aminotransferase (ALT) and aspartate aminotransferase (AST) were also determined in the third trimester of pregnancy.

Exosomes were concentrated from 500 µL of serum collected from the two experimental groups. The serum samples were centrifuged at 2000× *g* for 10 min at room temperature and the supernatant was collected for another 10,000× *g* centrifugation step for 30 min at room temperature. Microfiltration was thereafter pursued using 0.22 µm cellulose filters (Teknokroma, Barcelona, Spain) to discard the extracellular vesicles > 200 nm in diameter. Exosome concentration was performed according to the manufacturer’s instructions of the miRCURY Exosome Serum/Plasma Kit (Qiagen, Hilden, Germany). Thus, 200 µL of precipitation buffer were added to each of the samples, followed by an incubation step at 4 °C for 60 min and a room temperature centrifugation at 1500× *g* for 30 min. Thereafter, the supernatant was removed, and 270 µL resuspension buffer were added. Prior to exosome lysis, 15 µL sample aliquots were diluted in 1 mL of dd water for exosome size and zeta potential determination. Protein concentration was measured after exosome lysis using the Pierce BCA Protein Assay Kit (Thermo Fisher Scientific, Rockford, IL, USA) according to the manufacturer’s instructions.

The size of exosomes was determined by dynamic light scattering (DLS) method on a Zetasizer Nano ZS (ZEN 3600), (Malvern Instruments, Malvern, UK). The system measured the scattered light intensities at an angle of 173°, using water as the dispersant, at 25 °C. Afterwards, the Universal Dip Cell (ZEN1002) was used for zeta potential measurements by immersion into the sample. The results were processed and analyzed using the build-in Zetasizer Software 7.12 (Malvern Instruments, Malvern, UK).

### 4.4. Sample Preparation and Nano Liquid Chromatography—Mass Spectrometry Analysis

The concentrated exosomes were homogenized in a lysis buffer containing 1% DOC and 100 mM Tris-HCl (pH 7.5) using a rotor-stator mechanical homogenizer, on ice (Polytron PT 1300D, Kinematica, Malters, Switzerland). The protein rich supernatant was separated from the vesicles’ debris following a powerful centrifugation (20,000× *g*, 20 min, 4 °C). Protein quantification was performed using the Pierce BCA Protein Assay (Thermo Fisher Scientific, Rockford, IL, USA). Then, 50 µg of protein from each sample were purified by acetone precipitation, using a 1:4 volume ratio (protein to acetone, *v*/*v*). Cysteine carbamidomethylation was performed with a reducing buffer containing 8 M urea, 0.1 M Tris-HCl, 0.1 mM EDTA and 20 mM dithiothreitol, pH 8.8, followed by an alkylation buffer (80 mM iodoacetamide in 0.1 M Tris-HCl and 0.1 mM EDTA). A buffer containing 80 mM N-acetyl cysteine in 0.1 M Tris-HCl and 0.1 mM EDTA was used to quench the excess iodoacetamide from the sample. Urea dilution to <1 M, sodium deoxycholate addition (1% final concentration) and pH adjustment (~8.5) using 1 M ammonium bicarbonate were performed before the overnight proteolysis (1:50 enzyme to substrate ratio). Formic acid was used after ~16 h incubation to shift the pH to 2.5, for trypsin inactivation and deoxycholate precipitation, which was discarded following twice 20 min, 20,000× *g* centrifugation. C18 sorbent columns (Sep-Pak, Waters, Wexford, Ireland) were employed for peptide desalting which were thereafter vacuum dried and resuspended in 5% acetonitrile, 0.1% formic acid-containing buffer. For peptide quantity normalization, 280 nm absorbance measurements using the Pherastar FS system (BMG Labtech, Ortenberg, Germany) were performed.

The Easy nLC II nano-chromatographic system was coupled to the LTQ Orbitrap Velos Pro hybrid mass spectrometer (Thermo Scientific, San Jose, CA, USA) for peptide separation and mass spectra acquisition. For each sample, 1 µg of peptides in triplicate was separated in a 15 cm × 75 μm.d., C18, 3 μm, 120 Å nano-column (Thermo Fisher Scientific, Rockford, IL, USA) and the separation was performed using a 90 min, 3%–25% solvent B (acetonitrile with 0.1% (*v*/*v*) formic acid) over A (water with 0.1% (*v*/*v*) formic acid) solvent gradient at a flow rate of 300 nL/min. The MS was operated in a top 15 data-dependent configuration at 60 k resolving power for the full scan (350–1700 *m*/*z* domain) and collision induced dissociation (CID) fragmentation mode for MS2. Internal calibration was performed using the 445.120028 Da polysiloxane peak.

The raw files were processed for protein inference using Sequest HT search algorithm inside Proteome Discoverer 2.4 software (Thermo Fisher Scientific, Rockford, IL, USA). Uniprot/SwissProt Homo sapiens reference proteome was used (SwissProt TaxID *=* 9606, v 2019-10-04), with methionine oxidation set as a dynamic modification and cysteine carbamidomethylation as a static one, while a maximum of two missed cleavages was allowed. A reverse database search was performed for strict protein and peptide FDR settings (<5%). An in-house contaminant database was used to recognize and filter out common protein contaminants. Label free relative quantification was performed using the precursor ion quantifier node and was based on the intensity of the unique peptide precursors from 90% of replicate features. Normalization was performed on total peptide amount. Protein abundances were calculated as the average of the most abundant distinct peptide groups, while the protein ratio was directly calculated from the grouped protein abundances. The statistical significance of the quantification ratio comparison was calculated using the ANOVA hypothesis test. STRING bioinformatic freeware (v.11.0) was used for creating interaction-based networks and accession to KEGG (Kyoto Encyclopedia of Genes and Genomes) and Gene Ontology databases.

### 4.5. Western Blot Analysis

Twenty-five microliters of exosome homogenate were separated in Tris SDS 12% polyacrylamide gels, and transferred onto nitrocellulose membranes (Bio-Rad Laboratories, Hercules, CA, USA). Blots were blocked with 2% BSA in TBS and probed overnight at 4 °C with primary antibodies against CD9 (Thermo Fisher, Rockford, IL, USA, #MA1-80307), CD63 (Abcam, Cambridge, UK, ab193349), Alpha-1-B Glycoprotein A1BG (ThermoFisher, Rockford, IL, USA, # PA5-75479) and Haptoglobin-related protein HPR (Thermo Fisher, Rockford, IL, USA, # PA5-42206) followed by appropriate horseradish peroxidase (HRP)-conjugated secondary antibody (dilution 1:2000). The immune complexes were detected by enhanced chemiluminescence reaction using a digital detection system (ImageQuant LAS 4000, GE Healthcare, Chicago, IL, USA). All original blot images can be found in the Supplementary Material.

### 4.6. Statistical Analysis

For mass spectrometric bioinformatics analysis, statistical algorithms were employed for correcting the ANOVA-obtained p-values using the Benjamini–Hochberg algorithm based on the false discovery rate (FDR). For the other experiments, the data were expressed as the mean ± standard deviation (SD). Pearson’s correlation analysis was performed on patients’ characteristics associated with GDM and serum level parameters. Statistical analysis was performed by *t*-test; *p <* 0.05 was regarded as significant difference.

## 5. Conclusions

Understanding the pathophysiology of GDM is of great importance, given the high risk for both mother and child of development of type 2 diabetes and cardiovascular diseases later in life. The complement system plays an active role in metabolic disorders, adaptive immune response, homeostasis of host cells and removal of apoptotic cells—processes that are dysregulated in GDM. This is the first study to analyze CCC signaling pathway in serum exosomes in GDM pregnancies. We found decreased levels of Complement C3 (C3), Complement C5 (C5), C4-B (C4B), C4b-binding protein beta chain (C4BPB) and C4b-binding protein alpha chain (C4BPA), while C7 and C9 were upregulated. The serum exosomal levels of F12 were increased in the GDM group, which could help to explain one of the underlying complex mechanisms that lead to GDM. In addition, F12 inhibitors could prevent the adverse outcomes of GDM. Our data confirmed that increase of insulin release may lead to endothelial cell damage, with subsequent abnormal levels of VWF and AT III. Elevated expression of VWF and reduced expression of AT in plasma suggest endothelial injury and endothelial dysfunction. In our hands, the serum exosomal levels of VWF were upregulated in GDM pregnancies compared to normal pregnancies. These results open up the possibility of more prospective studies with larger cohorts to confirm the role of F12 in the pathogenesis of GDM and its complications.

In GDM, the impaired glucose uptake and insulin resistance is caused also by the disturbance of lipid transport and metabolism, in which apolipoproteins play an important role. Our study is the first one to analyze apoC-II in serum exosomes of GDM subjects. We documented that serum exosomal levels of apoC-II were higher in GDM pregnancies compared to normal pregnancies and are positively correlated with adiponectine and fasting glycemia. Apolipoproteins affect the activity of the complement system and we found that numerous complement proteins are positively associated with apoC-II protein. In parallel with the contradictory literature data regarding the statistical association of different clinical parameters and GDM, we identified interesting correlations that can help future studies to clarify the physiopathology of GDM. More studies are necessary to elucidate the interconnection between lipid metabolism and complement system in the pathophysiology of GDM.

The main limitation of the study is the relatively small number of biological replicates for the two conditions, which might have affected the robustness of the results. However, the conclusions of the study are based on biological trends supported by consistent up- or down-regulation of multiple proteins involved in the same processes, and are in line with previously published data. Validation on larger cohorts of patients is needed to increase the statistical power of our results.

In short, the original data resulting from this study demonstrate that:

1. Gestational diabetes regulates exosomal protein abundance associated with the complement and coagulation cascade;
2. Proteins involved in the thrombotic mechanism and cholesterol metabolism are differentially altered by the pathological state;
3. Statistical correlations have been obtained between the clinical and paraclinical parameters and the exosome proteome that could improve the understanding of gestational diabetes mellitus pathogenesis and evolution.

## Figures and Tables

**Table 1:** Clinical characteristics of the enrolled patients and subjects. Mother’s age (years); pre-pregnancy BMI (kg/m^2^); gestational age BMI at the moment at which GDM was diagnosed (kg/m^2^); serum concentrations of adiponectin (ng/mL), insulin (mU/mL), peptide C (ng/mL), proinsulin (pmol/L) and calculated steady-state β-cell function (%B), insulin sensitivity (%S) and homeostatic model assessment (HOMA)-insulin resistance (IR). Oral glucose tolerance test at 0 h (GTT 0 h), 1 h (GTT 1 h) and 2 h (GTT 2 h) during the third trimester of pregnancy, serum levels of creatinine, cholesterol, high density lipoprotein cholesterol (HDL), triglycerides (Tg) and uric acid all expressed as mg/dL; alanine aminotransferase (ALT) and aspartate aminotransferase (AST) both expressed as Ui/L, measured in the third trimester at time when GDM was diagnosed, glycated hemoglobin HbA1c (%); hemoglobin (g/dL); glutamic acid decarboxylase autoantibodies—antiGAD (iE/mL) and mean platelet volume (MPV). Data are expressed as means ± standard deviation (SD); * *p* ˂ 0.05, ** *p* ˂ 0.01; *** *p* ˂ 0.001.

Maternal Parameters	CG (*n* = 8)	GDM (*n* = 10)
Age (years)	26 ± 2.33	31.52 ± 4.24 ***
Pre-pregnancy BMI (kg/m^2^)	24.21 ± 5.13	25.99 ± 5.25
Gestational age BMI (kg/m^2^)	27.69 ± 5.28	29.93 ± 5.22
Adiponectin (ng/mL)	13.5 ± 5.78	9.17 ± 4.71 **
Insulin (mU/mL)	18.6 ± 12.05	12.99 ± 5.50 *
Peptide C (ng/mL)	9.70 ± 2.49	6.67 ± 4.64 *
Proinsulin (pmol/L)	3.09 ± 1.80	2.27 ± 0.60 *
Leptin (ng/mL)	14.30 ± 8.04	22.62 ± 10.74 *
Steady-state β-cell function (%B)	254.83 ± 98.68	145.56 ± 56.79 ***
Insulin sensitivity (%S)	55.5 ± 32.99	61.49 ± 25.59
HOMA-IR	2.43 ± 1.40	2.08 ± 1.33
GTT 0 h (mg/dL)	75.25 ± 7.20	94.09 ± 13.48 ***
GTT 1 h (mg/dL)	115.17 ± 19.86	188.04 ± 36.46 ***
GTT 2 h (mg/dl)	81.81 ± 23.32	153.67 ± 30.98 ***
Creatinine (mg/dL)	0.42 ± 0.14	0.47 ± 0.08
Total cholesterol (mg/dL)	253.73 ± 43.94	247.95 ± 39.29
HDL (mg/dL)	75.827 ± 23.67	73.75 ± 17.70
Tg (mg/dL)	146.29 ± 29.09	229.35 ± 75.37 *
Uric acid (mg/dL)	3.26 ± 0.75	3.76 ± 1.34
ALT (Ui/L)	15.176 ± 7.96	16.04 ± 12.66
AST (Ui/L)	19.49 ± 9.63	15.49 ± 6.29
HbA1c (%)	5.2 ± 0.17	5.453 ± 0.38
Hemoglobin (g/dL)	11.13 ± 0.82	11.02 ± 0.93
antiGAD (iE/mL)	0.21 ± 0.16	0.22 ± 0.19
MPV	10.76 ± 1.02	10.68 ± 1.50

**Table 2:** List of differentially abundant proteins that were bioinformatically associated with complement and coagulation cascades (CCC), platelet degranulation (PD), cholesterol metabolism (CM) pathways and thrombotic factors (TF). The Uniprot accession code together with inference (Sequest Score, number of unique peptides) are also listed.

Accession	Description	Gene	KEGG Signaling Pathway	Sequest Score	No. Unique Peptides
P01009	Alpha-1-antitrypsin	SERPINA1	PD, CCC	10,389.25	40
P04217	Alpha-1B-glycoproteinX9	A1BG	PD	1941.13	26
P08697	Alpha-2-antiplasmin	SERPINF2	PD, CCC	292.54	7
P02765	Alpha-2-HS-glycoprotein	AHSG	PD	1282.95	13
P01008	Antithrombin-III (AT3)	SERPINC1	PD, PF, CCC	1037.25	15
P04114	Apolipoprotein B-100	APOB	CM	49,878.59	368
P02655	Apolipoprotein C-II	APOC2	CM	665.5	6
P08519	Apolipoprotein(a)	LPA	CM	955.82	26
P02749	Beta-2-glycoprotein	APOH	CM, PD	1546.03	16
P04003	C4b-binding protein alpha chain	C4BPA	CCC	5665.54	41
P20851	C4b-binding protein beta chain	C4BPB	CCC	454.47	4
P00748	Coagulation factor XII (F12)	F12	CCC	165.39	7
P02745	Complement C1q subcomponent subunit A	C1QA	CCC	296.4	4
P02746	Complement C1q subcomponent subunit B	C1QB	CCC	828.78	7
P02747	Complement C1q subcomponent subunit C	C1QC	CCC	629.95	5
P00736	Complement C1r subcomponent	C1R	CCC	1981.15	30
P09871	Complement C1s subcomponent	C1S	CCC	2550.88	25
P01024	Complement C3	C3	PD, CCC	53,889.17	188
P0C0L5	Complement C4-B	C4B	CCC	21,108.1	4
P01031	Complement C5	C5	CCC	6026.05	69
P13671	Complement component C6	C6	CCC	1606.19	27
P10643	Complement component C7	C7	CCC	1264.74	23
P07358	Complement component C8 beta chain	C8B	CCC	1215.3	22
P02748	Complement component C9	C9	CCC	377.48	13
P05156	Complement factor I	CFI	CCC	780.32	18
P02751	Fibronectin	FN1	PD	6931.28	89
Q08380	Galectin-3-binding protein	LGALS3BP	PD	1342.04	24
P00739-1	Haptoglobin-related protein	HPR	TF	2730.7	9
P05546	Heparin cofactor 2 (HCII)	SERPIND1	CCC	843.94	17
P04196	Histidine-rich glycoprotein	HRG	PD	929.64	17
Q06033	Inter-alpha-trypsin inhibitor heavy chain H3	ITIH3	PD	264.44	13
P04264	Keratin, type II cytoskeletal 1	KRT1	PD	3669.52	38
P05155	Plasma protease C1 inhibitor	SERPING1	PD, CCC	375.04	9
P00747	Plasminogen	PLG	PD, TF, CCC	5912.95	70
P02768	Serum albumin	ALB	PD	69,845.72	115
P02766	Transthyretin	TTR	PD	2411.64	13
P07225	Vitamin K-dependent protein S	PROS1	PD, CCC	531.32	21
P04275	Von Willebrand factor	VWF	PD, TF, CCC	426.8	27

## Data Availability

The mass spectrometry data were deposited in the PRIDE [60,61] repository via ProteomeXchange [62] with the dataset identifier PXD035673.

## Supplementary Materials

The following supporting information can be downloaded at: https://www.mdpi.com/article/10.3390/molecules27175502/s1, Figure S1: Unaltered (uncropped and unadjusted) original images for CD63 (upper image set) and CD9 (lower image set) Western blot images for control (CG) and gestational diabetes mellitus. (GDM) samples, including the associated Ponceau S-colored membrane and protein marker, utilized for exosome-specific marker validation. In order to verify the specific first antibody signal, the membrane was cut in the middle of an additional control sample. The larger piece of membrane was incubated with both antibodies, while the smaller, cut, piece of membrane was incubated with only the secondary antibody. Figure S2: Unaltered (uncropped and unadjusted) original images for A1BG and HPR Western blot images, including the associated Ponceau S colored membrane and protein marker, utilized for relative quantitative information between the control (CG) and gestational diabetes mellitus. (GDM) samples. The upper side of the blot image was incubated with A1BG specific antibody, while the lower side was incubated with HPR corresponding antibody, followed by the appropriate secondary antibody. Figure S3: Dynamic light scattering measurements. Size (5 measurements) and zeta potential distribution (3 measurements) for representative samples.
