# Supplementary material for: Exosome Proteomics Reveals the Deregulation of Coagulation, Complement and Lipid Metabolism Proteins in Gestational Diabetes Mellitus
Source: Molecules. 2022 Aug 26;27(17):5502. doi: 10.3390/molecules27175502 (PMC9457917; doi:10.3390/molecules27175502)
Supplement: Supplementary file 1 [file molecules-27-05502-s001.zip › molecules-1872791-supplementary.pdf]

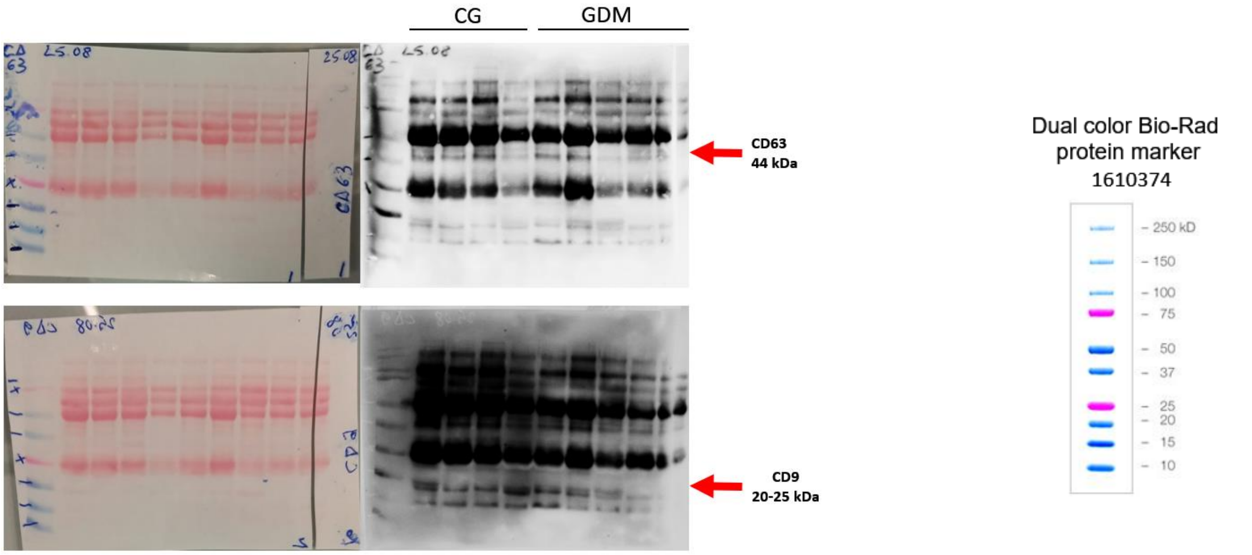

Figure S1: Unaltered (uncropped and unadjusted) original images for CD63 (upper image set) and CD9 (lower image set) Western blot images for control (CG) and gestational diabetes mellitus. (GDM) samples, including the associated Ponceau S-colored membrane and protein marker, utilized for exosome-specific marker validation. In order to verify the specific first antibody signal, the membrane was cut in the middle of an additional control sample. The larger piece of membrane was incubated with both antibodies, while the smaller, cut, piece of membrane was incubated with only the secondary antibody.

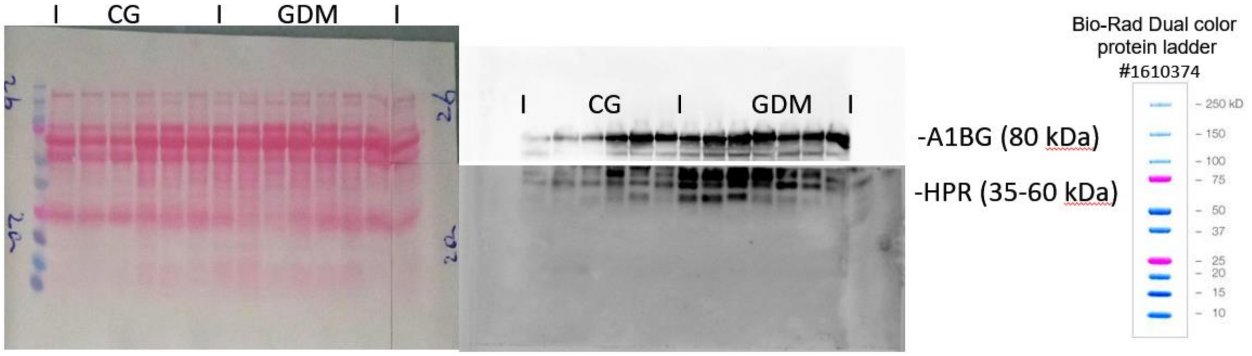

Figure S2: Unaltered (uncropped and unadjusted) original images for A1BG and HPR Western blot images, including the associated Ponceau S colored membrane and protein marker, utilized for relative quantitative information between the control (CG) and gestational diabetes mellitus. (GDM) samples. The upper side of the blot image was incubated with A1BG specific antibody, while the lower side was incubated with HPR corresponding antibody, followed by the appropriate secondary antibody.

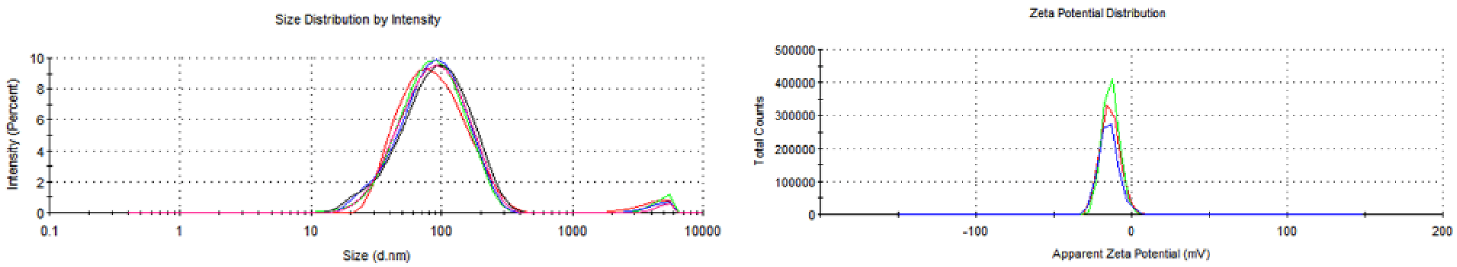

Figure S3: Dynamic light scattering measurements. Size (5 measurements) and zeta potential distribution (3 measurements) for representative samples.
